# The British Journals: Pathology, Practical Medicine, and Therapeutics

**Published:** 1840-01

**Authors:** 


### PATHOLOGY, PRACTICAL MEDICINE, AND THERAPEUTICS.

### *On Alkaline Indigestion.* By R. D. Thomson, M.D.

[Abstract of a paper read before the British Association at Birmingham.]

The author stated that he discovered this form of dyspepsia in 1835, and had communicated the results of his observations to the Medical Section of the British Association at Bristol. Since that period he had continued his researches, and had confirmed the accuracy of his first results, by the examination of a very large number of cases. It has been long known that in stomach complaints fluids are frequently ejected from that viscus into the mouth; and it has been by examining the chemical constitution of those fluids that the author has been enabled to simplify, in some considerable degree, one of the most disagreeable forms of dyspepsia. Dr. Thomson divides the fluids which he has detected in these complaints into *acid*, *alkaline*, and *neutral.*

1. The *acid* state is familiar to most persons. In the natural, there is no doubt that during a certain period of the process of digestion, the contents of the stomach exhibit an acid reaction; that is to say, that litmus paper, dipped in the fluid existing in the stomach, becomes red; that the fluid tastes acid, and that when distilled over, a quantity of pure water having been previously added, the fluid which passes into the receiver exhibits a faint acid reaction. This does not occur, according to Schultz, however, during the first half hour or hour of the process of digestion. The acid would, therefore, appear to be generated by the process. The discussion with respect to the nature of the acid, Dr. Thomson stated that he would reserve for the Chemical Section. When this natural acid, however, as it may be termed, accumulates to a certain extent, symptoms of disease exhibit themselves, in the form of a burning sensation at the pit of the stomach, with acid eructations, which do not, however, alleviate the pain. This is the characteristic symptom of *acid indigestion.*

2. The second form of indigestion, indicated by the fluid ejected from the stomach, Dr. Thomson terms *alkaline indigestion.* It is characterized by violent pain in the region of the stomach, accompanied, frequently, with headach and faintness, with a sensation of spasm or contraction in that viscus; the sensation goes on increasing, till it frequently becomes intolerable, and, at last, when the agony is complete, the patient is suddenly roused by a determination to the mouth of a large quantity of fluid, which must be immediately evacuated, to give place to a succession of similar occurrences; at last, however, the flow of fluid becomes so abundant as to constitute an actual stream; it continues to flow for some time, but gradually diminishes in quantity, and at length ceases, and with it the pain in the stomach. The latter is the characterizing symptom of *alkaline dyspepsia*, or *pyrosis*, as it has been frequently termed. But hitherto it has always been confounded with other forms of indigestion. Dr. Prout has published an account of his examination of the fluid of *pyrosis*, and has stated that it was acid; the fluid, however, was not procured by himself, but was sent him from one of the hospitals, where the mistake was very likely to occur. This form of indigestion occurs much more frequently than is generally imagined. Dr. Thomson stated, that out of forty or fifty patients daily seen at the Blenheim-street Dispensary, in London, he generally met with one or two affected with symptoms of this description. It frequently occurred in coincidence with affections of other organs—as of the uterus, liver, &c., and was often of such a pressing nature, that it required more of the skill of the medical man than the original disease. Certain it was, that it was absolutely necessary to treat it with as much care as the original complaint; and if the action had been allowed to go on for some time unchecked, the secondary affection became as firmly fixed as the original disease which had induced it; so that after the removal of the latter a second disease, as firmly rooted as the first, required to be taken under the physician's care: the treatment consisted of the administration of acids, tonics, and narcotics, which required to be prescribed with care, otherwise the *acid indigestion* was frequently induced, which was as difficult to eradicate as the alkaline form.

3. The last form of indigestion, as indicated by the fluid ejected by the mouth, which the author had met with, was a *neutral* state, which was of much rarer occurrence. Dr. Thomson had, however, met with several cases, and had succeeded in overcoming the disease by the use of tonics. *Lancet. Sept.* 21, 1839.

---

### *Observations on the Treatment of Acute Rheumatism by Opium.*

By D. J. Corrigan, m.d., Physician to Jervis-street Hospital.

[Ever since the publication of Dr. De Roches' paper on the subject, in the first Number of the Edinburgh Medical and Surgical Journal, (April, 1805,) we have been advocates for the use of opium in certain cases of acute rheumatism; and we could adduce some striking cases in favour of this treatment. Still, our experience does not justify our recommending it as always successful, or always the best practice. Dr. Corrigan's paper contains important additions to our previous experience. We can only find room for some of the practical remarks, and one of the eight cases recorded in it.]

The treatment by opium shortens the duration of the disease; it enables the patient to pass through so painful a disease with comparatively little suffering; it husbands his strength, so that when convalescent he rises from bed with comparatively little depression of vascular energy and muscular strength; and lastly, it diminishes very remarkably the tendency to the occurrence of such complications as pericarditis, endocarditis, &c.

The most important rule to be remembered in employing opium for the cure of acute rheumatism is, *that full and sufficient doses* shall be exhibited. I have heard of opiate treatment having disappointed some who have tried it. On enquiry, I have learned that in those cases it has been given only to the extent of a grain every fourth or every sixth hour. This is not "the treatment of rheumatism by opium;" it is making the patient worse than before; it is inflicting on the patient the mischief arising from the stimulant effects of the drug, and withholding from him all the benefits of its sedative influence. The opium should always be increased in dose, both in frequency and quantity, until the patient feels decided relief; and should be then kept up at that dose until the disease is steadily declining. The first indication that tells the practitioner he has reached the proper dose, is, the statement of the patient, who in reply to an enquiry as to how he has passed the night, probably says that he has not slept, but that he is free from pain and feels comfortable. This effect having been attained, the opium may then be continued in repetitions of the same dose as to frequency and quantity.

I think about ten or twelve grains in every twenty-four hours, will be found the average quantity required. The tolerance of the remedy is a remarkable feature in the treatment, and may, I think, be fairly adduced as an argument in favour of its propriety. The head is not affected by the large quantity of opium administered. This is remarkably shown in Dr. Aldridge's case, where the head was not injured by the opium, even though there had been previously a tendency to derangement of cerebral functions in all previous febrile affections. There is another singular circumstance connected with the exhibition of the opium. It is the occurrence of diarrhœa, while the patient is using the opium even in full doses; in some instances, the diarrhœa becoming so troublesome as to require starch enemata, or chalk mixture, with kino. It is seldom necessary to purge the patient while administering the opium; indeed the pains are sometimes brought back by the administration of a purgative, either from the patient catching cold in rising from bed, or from the irritability produced by the action of the purgative. The patient's bowels, if they have not been constipated in the commencement of the attack, may be not only safely, but with benefit, not disturbed more than once in two days.

CASE VI.—On Wednesday, 24th April, I visited my friend Dr. Aldridge, in a very severe attack of the disease. Nearly all the large joints were swollen and acutely painful, and the pains were shifting from joint to joint. The pulse was 120; the tongue was very foul, but moist: the want of rest, from the agony of the pains, was most distressing. The attack was of three days' duration when I first saw him. I immediately put him on the opiate treatment; he first got one grain every two hours; the quantity was then increased to a grain every hour; and this was continued for thirteen days, with the administration of an occasional purgative. On the fourteenth day he began to take the mist. guaiaci c. sulph. quinæ; and on the fifteenth day he was walking about his parlour, complaining only of not being as strong as usual, but free from pain and swelling; and he described the treatment as most grateful to his feelings, the pains being so much lulled by the opium, that he passed through the attack with very little suffering. In reply to some inquiries of mine, as to his impressions relative to the opiate plan of treatment, he favoured me with the following:

"*Mount Michael; Glasnevin.*

"Dear Doctor,—You ask me for my recollections of rheumatism; they are not very agreeable, but I owe you the performance of a task, more disagreeable than that of recalling the remembrance, as some return for the comfort your treatment afforded me when labouring under the reality.

"I suffered eight years previously, under two successive attacks of rheumatic fever, from each of which my recovery was tedious, extending through a period of some months.

"During the month of April this year, my strength was much exhausted by the exertions of the preceding winter. I was low-spirited, weak, and hypochondriac. The first warning of a coming attack consisted in a sensation of occasional faintness; a feeling of uneasiness about the region of the heart, as if its throbbings were about to cease; then came, during the two following days, coldness, shiverings, general soreness, and the other agreeable forerunners of fever. I had recourse to my usual panacea for all ills—a warm bath—but found myself much worse after it. Acute pains began to nestle themselves, particularly in the small joints. The next morning I drew some blood from my arm; but getting no relief, I requested that evening your attendance.

"You came, and found my ankles and wrists swollen enormously; while I writhed under a succession of exquisite aches, now in one joint, now in another, to which the pain of toothach is elysium. You afforded me, however, some comfort by telling me my heart was untouched; and the primæ viæ requiring to be cleansed, you directed me to take some purgative medicine; which having performed its office, you desired me to commence taking one grain of opium every second hour.

"I confess that I was somewhat afraid of what appeared to me very large doses of this powerful drug, especially as my head always had a tendency to be affected whenever I had fever of any kind. It was therefore with some misgiving I obeyed you, but soon had reason to congratulate myself on the effects of your advice, for during the remainder of my illness, i.e. from the second day after being forced to succumb, the pains, although they visited me occasionally, were by no means so intolerable; I slept much, my intellect remained clear, except when occasionally I took an overdose of the opium, (for as soon as I began to experience its good effects, I became quite enamoured of it,) and, in fine, I was enabled to walk down stairs the fourteenth day after taking to bed. During another week, I rubbed such joints as were occasionally painful with a liniment made with sulphur and camphorated oil, and took internally quinine and guaiacum; but since then, now during a period of four months, I have not had the slightest return of the disease. As nearly as I can recollect, I swallowed during my illness about two hundred grains of opium.

"Such are my reminiscences of rheumatism. To the influence of large and repeated doses of opium in relieving its agonies and shortening its duration, I gladly and gratefully bear testimony; but I must confess, that whatever may be my veneration for the 'lights of science,' I have not the slightest ambition of again personally testing its efficacy.

"I remain, &c. &c., J. Aldridge."

*Dublin Journal. Nov.* 1839.

---

*Outlines of a Case of Acute Anasarca and Convulsion after Scarlatina.*
By Marshall Hall, m.d., f.r.s., &c.

The patient was a boy, aged twelve. Sixteen days before, he had gone through scarlatina in its very mildest form; he had scarcely been confined to bed, and had not suffered from the nimia medici diligentia; he had appeared quite well. On this Sunday morning he was seized with swelling of the face, which came on and increased equally suddenly and rapidly. With this symptom there was the appearance of sudden and serious collapse, and soon afterwards convulsions followed by coma.

When I first saw my patient, there were convulsions followed by deep coma; wine and brandy on the table indicated sufficiently the previous state of the case. I felt persuaded, in spite of these appearances, that the only hope was afforded by relieving the vascular system within the head, and yet that the measure was not unattended with danger. This view was freely explained to the boy's father, who very sensibly said, he confided his son's life to the hands of his medical advisers.

We placed the patient upright, and Mr. Duffin opened the jugular vein. I kept my finger on the pulse, whilst we allowed twenty ounces of blood to flow! The convulsions ceased, and the coma diminished, but did not disappear. We then ventured to open a vein in the arm, and abstracted seven more ounces of blood! In less than an hour, the little patient knew his parents. We prescribed calomel and purgative medicine; a cold lotion to the head, and fomentations to the feet; afterwards leeches were applied; but the bloodletting was *the* remedy to which the amendment was obviously due. The little boy recovered forthwith, and what is important, without the least symptom of the morbid effects of loss of blood.

I must here remark, 1st, that acute anasarca and convulsions may, and frequently do, follow the mildest cases of scarlatina, perhaps because, after such cases, less precaution is usually taken to clear the bowels; 2d, that in all cases of acute anasarca there is the danger of an affection of the head, arachnitis, coma, or convulsion; and 3d, that in such cases the remedy is bloodletting,—bloodletting until relief and security are obtained. *Lancet. Nov.* 30, 1839.

---

*Clinical Remarks on some Cases of Liver Abscess presenting externally.*
By J. G. Malcolmson, m.d.

We recommend the perusal of this paper to all our brother practitioners in India. It contains many valuable observations on the pathology and treatment of one of the most frequent and fatal diseases of that country. The principal objects of Dr. Malcolmson are, to point out the not unfrequent occurrence of gangrene in cases of hepatic abscess, when opened by the surgeon; and to enter his protest against the very injurious practice, so prevalent of late years, of giving calomel in too large doses, and under circumstances when its action is most injurious, as when abscess is fully formed. Dr. M. also clearly shows that this practice, as also the equally injurious one of extreme depletion by the lancet, was too common with practitioners in India, in the early part of the present century. *Edinburgh Journal. October,* 1839.

